# Natural Ghee Enhances the Biochemical and Immunohistochemical Reproductive Performance of Female Rabbits

**DOI:** 10.3390/life13010080

**Published:** 2022-12-27

**Authors:** Hassan T. El-Gharrawy, Kadry M. Sadek, Sahar F. Mahmoud, Attaa. M. Abd Elrehim, Mustafa Shukry, Heba I. Ghamry, Samah F. Ibrahim, Liana Fericean, Mohamed Abdo, Mohamed M. Zeweil

**Affiliations:** 1 Department of Biochemistry, Faculty of Veterinary Medicine, Damanhour University, Damanhour 22511, Egypt; 2 Department of Histology, Faculty of Veterinary Medicine, Damanhour University, Damanhour 22511, Egypt; 3 Department of Physiology, Faculty of Veterinary Medicine, Damanhour University, Damanhour 22511, Egypt; 4 Department of Physiology, Faculty of Veterinary Medicine, Kafrelsheikh University, Kafrelsheikh 33516, Egypt; 5 Department of Home Economics, College of Home Economics, King Khalid University, P.O. Box 960, Abha 61421, Saudi Arabia; 6 Department of Clinical Sciences, College of Medicine, Princess Nourah bint Abdulrahman University, P.O. Box 84428, Riyadh 11671, Saudi Arabia; 7 Department of Biology and Plant protection, Faculty of Agriculture, University of Life Sciences “King Michael I”, 300645 Timișoara, Romania; 8 Department of Animal Histology and Anatomy, School of Veterinary Medicine, Badr University in Cairo (BUC), Cairo 11829, Egypt; 9 Department of Anatomy and Embryology, Faculty of Veterinary Medicine, University of Sadat City, Sadat City 32897, Egypt

**Keywords:** ghee, margarine, fertility, antioxidants, reproductive hormones

## Abstract

The reproductive effects of several dietary fats (margarine, ghee, and olive oil) on female rabbits were studied. For that purpose, 40 mature female rabbits were designed into four groups of ten rabbits each. Group I was given a control diet, Group II received 10% margarine, Group III received 10% ghee, and Group IV received 10% olive oil; after two months, all rabbits were sacrificed. Lipid profile and reproductive hormones levels were assayed in serum besides ovarian antioxidant enzyme and lipid peroxidation. Furthermore, ovarian tissue was examined using hematoxylin–eosin staining and immunohistochemistry of estrogen, follicle-stimulating hormone (FSH), luteinizing hormone (LH) receptor, and caspase 3. Our data revealed that the margarine significantly (*p* < 0.05) increased lipid profile and malondialdehyde (MDA) level, which decreased in olive oil and ghee compared to the control. In addition, serum FSH and estrogen (estradiol (E2)) were significantly (*p* < 0.05) decreased in the group treated with margarine. Furthermore, there was a significant decrease in ovarian superoxide dismutase (SOD) and catalase activity in the margarine-treated group. In contrast, SOD and MDA showed a significant (*p* > 0.05) increase in the olive oil and ghee- treated group compared to the control group. At the same time, there was a significant increase in serum FSH and (estradiol (E2)) in the ghee and olive oil groups, respectively, compared to the control. The margarine feed group showed moderate immunoreaction of estrogen, FSH, LH receptor, and strong caspase 3, while ghee and olive oil showed strong immunoreaction of estrogen, FSH, LH receptor, and mild immunoreaction of caspase 3 in ovarian tissue. Photomicrograph of rabbit ovarian tissue showed vacuolation in small and growing follicles in the margarine group but appeared normal in ghee and the olive oil-treated group. In conclusion, based on these results, olive oil and ghee have a strong capability of enhancing lipid profile, antioxidant status, and female hormonal functions.

## 1. Introduction

Lipids (fats and oil) are a vital source of energy and nourishment in human health and are a substantial component of our daily diet. Lipids have a variety of biological tasks, including producing vital fatty acids, storing and transferring metabolic fuels, preserving organisms’ surfaces, forming cellular membranes, and assisting cell identification [1].

Fats and oil may be of vegetable, animal, and marine origins. Vegetable fats include olive oil, sunflower, corn, and soybean oil. Usually, common vegetable oils, including olive, soybean, sesame, and sunflower, are low in saturated fats, but palm oil, palm kernel oil, and coconut oil are high in saturated fats [2].

Olive oil is produced from the olive tree (*Olea Europaea*), a classic Mediterranean tree crop; it is added to food, pharmaceuticals, and skin care items because of the antioxidant capacity of its polyphenols, olive oil possesses anti-inflammatory, anti-thrombotic, antihypertensive, antioxidant, and vasodilator properties [3]. Moreover, it has a larger concentration of monounsaturated fatty acids, which reduces free radical levels by scavenging them and inhibits the oxidation of low-density lipoprotein (LDL) [4].

Partially hydrogenated vegetable oils (margarine) are utilized in the food sector to increase the palatability of baked goods and sweets. Because of its extended shelf life, suitability for deep frying, and semi-firmness. During the process of hydrogenation, some unsaturated fatty acids’ double bonds can be isomerized and changed from the cis to the trans-state. TFAs have negative health consequences and can induce various disorders [5]. Including an increased lipid profile, risk of coronary heart disease [6,7,8], insulin resistance, and dyslipidemia [9].

Ghee is a dairy product made from cattle, sheep, goat milk, cream, or butter high in saturated fatty acids (SFA) [10]. Ghee is mostly composed of fatty acids, cholesterol, omega, saturated fats, monosaturated fats, polyunsaturated fats, and vitamins A, B, D, K, and E; it also contains very little water [11]. Additionally, it enhances lipid profiles, antiatherogenic potency, and antioxidant status [12], and has positive effects on the reproductive system [13].

Nutrition and reproduction have long been interconnected since an animal’s ability to reproduce depends on its nutritional state. This connection has been studied in research throughout the years, frequently by changing diets in different ways and tracking changes in reproductive characteristics. Adding fat to the diet is one of the most important dietary adjustments that can affect the reproductive system [14]. Previous reproductive studies showed an increase in the number and diameter of ovarian follicles in the fat-supplemented group [15].

Dietary lipids change human fertility, according to studies on the impact of dietary fat on human reproduction. They can change sex hormones and impact men’s and women’s fertility [16,17].

Therefore, it is necessary to evaluate the effect of margarine, ghee, and olive oil consumption on the oxidant and antioxidant markers, fertility, reproductive hormones, and histopathological changes in female rabbits.

## 2. Materials and Methods

### 2.1. Experimental Animals

The present study was conducted on 40 white female New Zealand rabbits aged six months, weighing about 2.5 kg, purchased from the Agriculture Faculty, University of Alexandria, Egypt. The chosen animals were housed individually in standard dimension wired metallic cages (35 × 35 × 60 cm) at room temperature as well as under good ventilation and received water ad libitum and a standard balanced diet (Table 1) for one weeks before the start of the experiment for acclimatization and to ensure normal growth and behavior as well as exclude any diseased rabbit. All of the research protocols followed the European Community Directive (86/609/EEC) and the national rules on animal care following the NIH Guidelines for the Care and Use of Laboratory Animals (8th edition). They were approved by the Institutional Animal Ethics Committee guidelines for animal care and use at the Faculty of Veterinary Medicine, Damanhour University (Approval Number: R/52).

The experimental period was extended to 2 months. Ingredients of the basal diet were as follow:

### 2.2. Dietary Fat Sources

Margarine, ghee, and olive oil were obtained from the local market in Behaira Governorate, Egypt (See Supplementary Table S1 for the margarine, butter, and ghee).

### 2.3. Experimental Design

The 40 rabbits were allocated to 4 groups, 10 rabbits each. The experimental diets were formulated to include margarine, ghee, and olive oil at 10% of the diet [18] for 2 months. Group I (control) rabbits received a basal diet, and Group II (margarine) received a 10% margarine and + 90% basal diet. Group III (ghee) received 10% ghee +90% basal diet, and Group IV (olive oil) received 10% olive oil + 90% basal diet. Food and water were available at all times.

### 2.4. Blood Sampling

At the end of the 8 weeks from the start of the experiment, using a sterilized disposable syringe and needle, blood samples were collected from the rabbit’s ear vein after fasting overnight. The blood was collected into a labeled clean, dry Wasserman tube and left for 30 min to coagulate at room temperature. It was centrifuged (KS-5000; Kubota, Tokyo, Japan) at 3000× *g* rpm for 15 min. The obtained serum samples were collected in clean sterilized rubber stopper glass vials and stored at −20 °C until used for biochemical analysis of serum lipid profile and female reproductive hormones.

### 2.5. Measuring Serum Biochemical Parameters

Triacylglycerol levels (TAG) were measured according to [19], and cholesterol levels were measured according to [20], using Boehringer Mannheim colorimetric kits (Mannheim, Germany). High-density lipoprotein (HDL) cholesterol (HDL-C) was also assessed and HDL concentrations were measured according to Lopes-Virella et al. [21] by using kits that BIOLABS SAS provided. One serum aliquot was precipitated with phosphotungstic acid and magnesium chloride; afterward, the cholesterol content was evaluated in the clear supernatant (Biolabo S.A., Maizy, France).

VLDL-c and LDL-c were calculated according to the formula of Tremblay et al. [22], Friedewald et al. [23]. VLDL-c = TAG/5 and LDL-c = Total Cholesterol − (HDL-c + VLDL-c). Serum Follicle-stimulating hormone (FSH), luteinizing hormone (LH) were by the Abcam Company [24]. The serum FSH (#MBS2502190), LH (#MBS729873), estrogen (#MBS9302240), and progesterone (#MBS762170) were determined by ELISA kits (My BioSource, San Diego, CA, USA) using a UV-vis spectrophotometer (Epoch Biotech, Winooski, VT, USA).

### 2.6. Ovarian Tissue Sampling

Tissue sampling for determination of oxidative stress and antioxidants assay. At the end of second month, Ovaries from rabbits (7 rabbits from each group) were dissected after an incision of the abdominal wall. The sacrificed rabbits were eviscerated, and the ovary wash vested from the carcass and divided into 3 parts; the first part was washed with phosphate-buffered saline (PBS) solution, PH 7.4 containing 0.16 mg/mL heparin to remove any red blood cells and clots. The ovarian tissue was homogenized in 5 mL cold PBS per gram tissue (1:5 dilution). All samples were centrifuged at 4000× *g* rpm for 15 min at 4 °C. The supernatant was collected and stored at −20 °C till biochemical analysis [25]; lipid peroxidation was assayed by determining the MDA level according to [26], ovarian superoxide dismutase (SOD), and catalase (CAT) enzyme activities were determined following Nishikimi et al. [27] and Aebi [28], respectively, that the Bio-diagnostic Company, Egypt, provided. The second part was used for ovarian tissue sampling for histological examination. 

It was fixed in 10% neutral-buffered formalin for 2–5 days and grossly examined for any pathological changes. The ovarian samples were dehydrated in ascending titer of ethyl alcohol from 50% to absolute. The clearance of the samples was applied using xylene (three changes), and then paraffin impregnation was done in the hot oven using melted paraffin wax (three changes) at 56 °C. Finally, blocks of the processed samples were prepared using paraffin wax and cut using a rotatory microtome. From the sample blocks, 5–7 µm-thick paraffin sections were obtained, mounted on egg albumin–glycerin coated glass slides, and dried in an electrical incubator for 30–60 min at 45 °C then stained with hematoxylin and eosin (H & E). The aforementioned histological stains were applied [29]. Examination of histological slides was done using light microscopy (Labo America, Inc. Fermont, CA, USA) supported with an ocular lens (x10, x20, and x40) and a digital camera for collecting photos.

The third part was used for ovarian immunohistochemistry: ovarian samples were fixed in 4% paraformaldehyde in PBS at pH 7.4 for 2 days at 4 °C, then embedded in paraffin blocks. Then using a microtome, samples were cut off to the desired thickness (3–5 µm) and mounted on positively charged gelatin-coated slides. The slides were then dried by incubation at 45 °C for a few hours and used for immunohistochemical localization of estrogen, FSH, LH receptor, and caspase 3. Immunostaining of paraffin-embedded ovarian samples was carried out as follows; inactivation of endogenous peroxidase using 0.3% H_2_O_2_ in methanol, and the autoclave antigen retrieval was made. The sections were blocked by PBS containing 5% bovine serum albumin for an hour and then incubated with primary antibody at 4 °C overnight in a humid chamber. After washing with PBS, sections were incubated for 30 minutes at RT with a biotinylated secondary antibody. ABC complex (Vector Laboratories, Burlingame, CA, USA) was then applied for an hour at RT, and the color was developed using a DAB solution (Sigma-Aldrich, St. Louis, MO, USA). Finally, the sections were counterstained with Mayer’s hematoxylin, washed with distilled water, air-dried, mounted with Entellan (Merck, Darmstadt, Germany), and photographed (Olympus, Tokyo, Japan). In the control experiments, the elimination of primary or secondary antibodies or ABC complex was performed therefore, all staining was abolished, and no positive signals could be determined.

### 2.7. Statistical Analysis

Data were analyzed by analysis of variance (one-way ANOVA) using the general linear model procedure of SPSS program version 20 (SPSS, Richmond, VA, USA). Variations among means were assessed using Tukey as a post hoc test.

## 3. Results

The biochemical effects of margarine, ghee, and olive oil on fertility in female rabbits will be summarized under the following headings: 

Effects on body weights. Margarine-fed rabbits had significantly (*p* < 0.05) increased body weights concerning control one. Ghee or olive oil-fed rabbits showed non-significant (*p* < 0.05) increased body weight compared to NC rabbits (Table 2).

Effects on oxidant and antioxidants: MDA, Catalase, and SOD.

Significantly higher (*p* < 0.05) ovarian MDA concentrations were in margarine-treated rabbits when compared to NC animals, while ghee and olive oil-treated rabbits showed significantly lower (*p* < 0.05) MDA levels than the margarine-treated rabbit’s group. Olive oil-treated rabbits showed significantly lower (*p* < 0.05) MDA levels than NC rabbits’ group. A significant decrease (*p* < 0.05) in catalase and SOD activities in margarine rabbits than the NC groups, while ghee and olive oil-treated rabbits showed a significant increase (*p* < 0.05) in catalase and SOD activities as compared to the margarine-treated rabbits’ group (Table 2).

### 3.1. Fertility Hormonal Effects

Significant higher (*p* < 0.05) serum FSH and estradiol (E2) and non-significant higher levels of LH, progesterone, and testosterone in ghee and olive oil-treated rabbits than NC groups, while margarine-treated group illustrated significantly lower (*p* < 0.05) serum FSH and estradiol (E2) and non-significant lower levels of LH, progesterone, and testosterone compared to NC groups. Serum prolactin levels were significantly increased in the margarine-treated group and non-significantly decreased in ghee and olive oil-treated rabbits compared to NC rabbits. Serum prolactin levels were significantly increased in the margarine-treated group compared to ghee and olive oil-treated rabbits (Table 3).

### 3.2. Effects on Lipid Profile, Hepatic (ALT), and Renal (Creatinine)

Significant cholesterol, low-density lipoprotein (LDL), very-low-density lipoprotein (VLDL), alanine transaminase (ALT), and creatinine increase in the margarine-treated group compared to the NC group. At the same time, there was a significant decrease in TAG, cholesterol, LDL, VLDL, ALT, and creatinine in the olive oil-treated group compared to the NC group. The ghee-treated group showed a significant decrease in TAG, a non-significant increase in cholesterol, LDL, ALT, and creatinine, and a non-significant decrease in HDL and VLDL compared to the NC group. Ghee and olive oil-treated groups showed a significant increase in HDL and a significant decrease in TAG, cholesterol, LDL, VLDL, ALT, and creatinine compared to the margarine-treated group, as shown in Table 4.

### 3.3. Histopathological Examination

**Figure 1:**
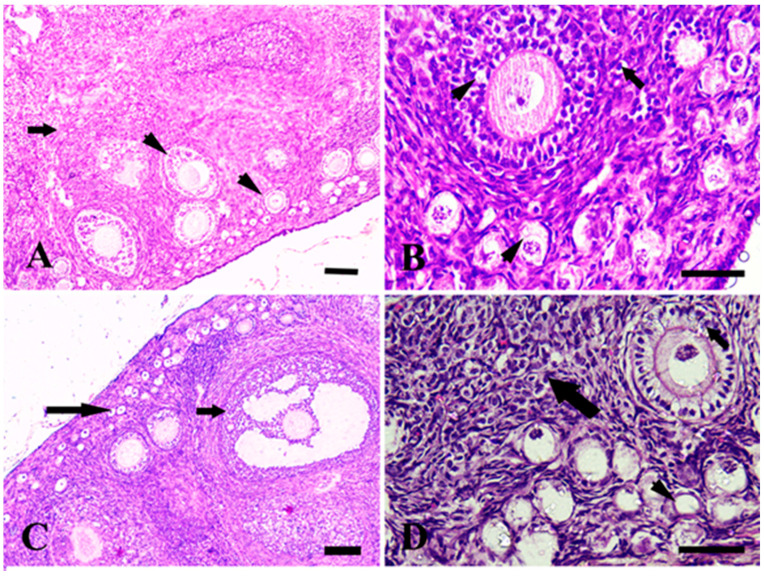
Photomicrograph of rabbit ovarian cortex stained by hematoxylin and eosin. (**A**) The control group with normal histological structure in small and growing follicles (head arrows) and surrounding stromal cells (arrow). (**B**) The margarine-treated group showed vacuolation in small and growing follicles (head arrows) and surrounding stromal cells (long arrow). (**C**) The ghee-treated group showed normal small follicular follicles (long arrow) and granulose cells of growing follicles (short arrow). (**D**) The olive oil-treated group showed proliferation in follicular cells of primary follicles (short arrow), small follicles (arrowhead), and stromal cells (long arrow). Scale bar = 100 µm in (**A**,**C**) and 50 µm in (**B**,**D**).

The ovaries are considered female gonads. The small follicles found at the periphery of the ovarian cortex and growing mature follicles were located deep within the cortex, as shown in Figure 1A. The margarine-treated group showed vacuolation in small and growing follicles and surrounding stromal cells (Figure 1B). The ghee-treated group showed normal small follicular follicles and granulosa cells of growing follicles (Figure 1C). The olive oil-treated group showed proliferation in follicular cells of primary follicles, small follicles, and stromal cells (Figure 1D).

### 3.4. Immunohistochemical Findings

**Figure 2:**
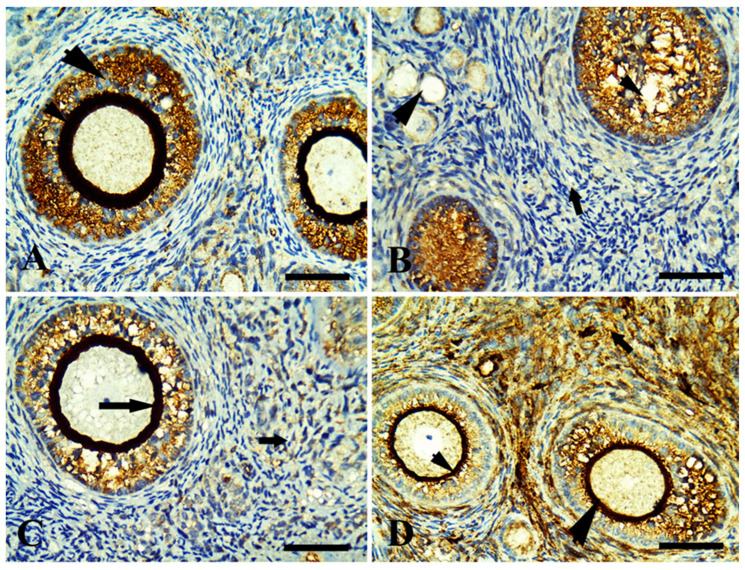
Immunohistochemically stained sections of rabbit ovarian cortex with estrogen receptor, scale bars = 50 µm. (**A**) control ovary showing positive immunoreaction in growing follicles (zona pellucida, thin arrowhead) and granulosa cells (thick arrowhead). (**B**) The margarine feed group showed mild immunoreaction in ovarian follicles with fibrotic granulosa cell (arrowhead) and mild in surrounding stromal cells (arrow). (**C**) The ghee-treated group showed intense immunoreaction in growing follicles, zona pellucida (long arrow), and moderate in stromal cells (short arrow). (**D**) The olive oil-treated group showed intense immunoreaction in growing follicles (zona pellucida; granulosa cells; arrows head) and stromal cells (long arrow).

**Figure 3:**
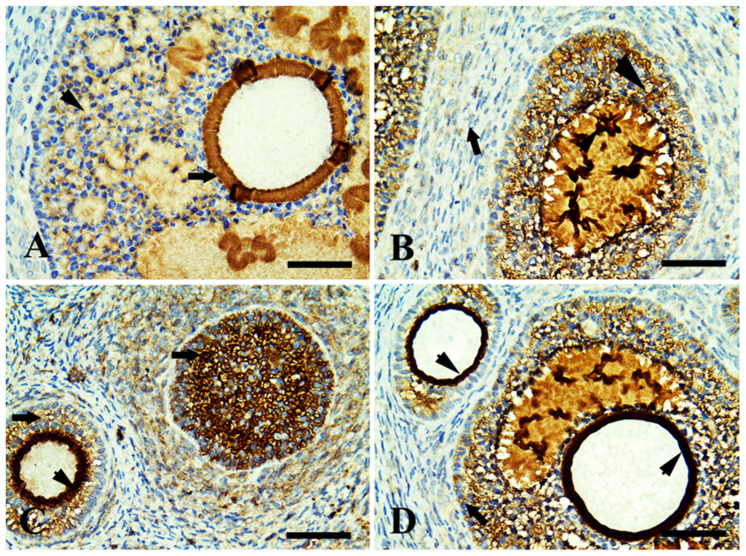
Immunohistochemically stained sections of rabbit ovarian cortex with follicle-stimulating hormone (FSH) receptor, scale bars = 50 µm. (**A**) control group showing positive immunoreaction in growing follicles (zona pellucida; long arrow) and granulosa cells (arrowhead). (**B**) The margarine-treated group showed mild immunoreaction of necrotic granulosa cells of the growing follicle (arrowhead) and no reactivity in surrounding stromal cells (long arrow). (**C**) The ghee feed group showed positive immunoreaction in growing follicles (granulosa cells; long arrows) and zona pellucida (arrowhead). (**D**) The olive oil feed group showing strong positive immunoreaction in growing follicles granulosa cells (long arrow) and intense immunoreaction (zona pellucida; arrows head).

**Figure 4:**
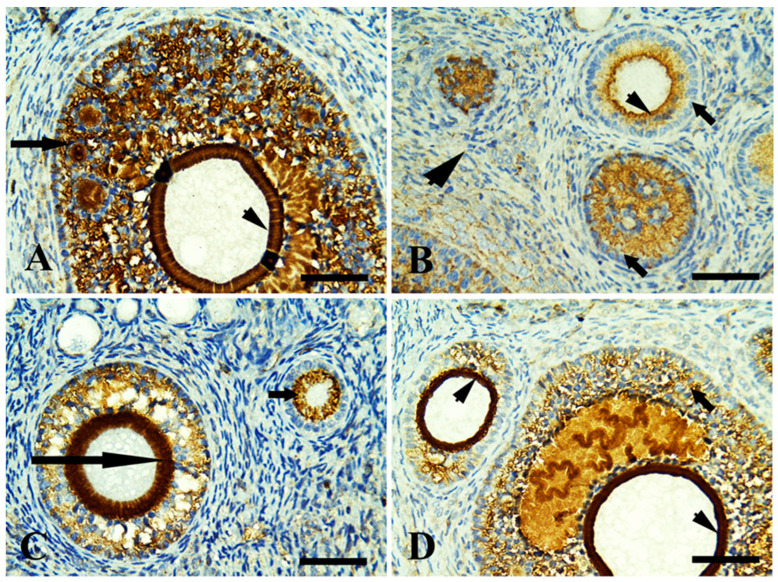
Immunohistochemically stained sections of rabbit ovarian cortex with luteinizing hormone (LH) receptor, scale bars = 50 µm. (**A**) The control group showed positive immunoreaction in large-growing follicles (zona pellucida, arrowhead) and granulosa cells (long arrow). (**B**) The margarine feed group showed mild immunoreaction in growing follicles in zona pellucida (arrowhead), no reactivity in granulosa cells (arrows), and surrounding stromal cells (thick arrowhead). (**C**) The ghee feed group showed strong immunoreaction in growing follicles (zona pellucida; long arrow) and moderate in granulosa cells (arrow). (**D**) The olive oil feed group showed strong immunoreaction in large antral follicles (zona pellucida; arrowheads) and moderate in granulosa cells (long arrow).

Control ovary showing positive immunoreaction in growing follicles only (zona pellucida and granulosa cells) in Figure 2A. The margarine feed group showed mild immunoreaction in ovarian follicles with fibrotic granulosa cells and was mild in surrounding stromal cells (Figure 2B). The ghee-treated group showed an intense immunoreaction in growing follicles, zona pellucida, and moderate in stromal cell (Figure 2C). The olive oil-treated group showed an intense immunoreaction in growing follicles (zona pellucida, granulosa cells) and stromal cells (Figure 2D). In relation to the FSH receptor that is necessary for follicular development, the control ovary showed positive immunoreaction in growing follicles in zona pellucida and granulosa cells (Figure 3A). The margarine-treated group showed a mild immunoreaction of necrotic granulosa cells of growing follicles and no reactivity in surrounding stromal cells (Figure 3B). The ghee feed group showed a positive immunoreaction in growing follicles (granulosa cells) and zona pellucida (Figure 3C). The olive oil feed group showed a strong positive immunoreaction in growing follicles granulosa cells and intense immunoreaction zona pellucida (Figure 3D). Follicular development depends on the luteinizing hormone receptor (LHR) that is located predominantly on ovarian thecal cells and stimulates ovarian androgens and steroid precursors production that are transported to granulosa cells for aromatization to estrogens. The control group showed a positive immunoreaction in large-growing follicles of zona pellucida and granulosa cells (Figure 4A). The margarine feed group showed a mild immunoreaction in growing follicles in (zona pellucida), and no reactivity in granulosa cells and surrounding stromal cells (Figure 4B). The ghee feed group showed strong immunoreaction in growing follicles (zona pellucida) and moderate in granulosa cells (Figure 4C). The olive oil feed group showed strong immunoreaction in large antral follicles (zona pellucida) and moderate in granulosa cells (Figure 4D). 

**Figure 5:**
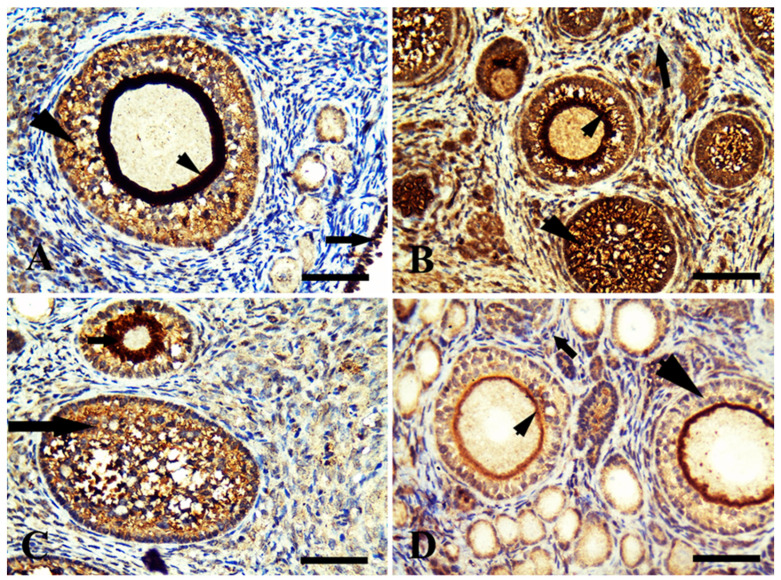
Immunohistochemically stained sections of rabbit ovarian cortex with Caspase 3, scale bars = 50 µm. (**A**) The control group showed positive immunoreaction in growing follicles (zona pellucida, arrowhead), some granulosa cells (thick arrowhead), and ovarian surface epithelium (long arrow), while no immunoreaction in surrounding stromal cells. (**B**) The margarine feed group showed strong immunoreaction in growing follicles (zona pellucida, thin arrowhead), granulosa cells (thick arrowhead), and stromal cells (long arrow). (**C**) The ghee-treated group showed moderate immunoreaction in growing follicles (granulosa cells, very long arrow), zona pellucida (long arrow), and mild in surrounding stromal cells. (**D**) The olive oil feed group showed mild immunoreaction in growing follicles (zona pellucida, arrowhead), granulosa cells (thick arrowhead), and stromal cells (long arrow).

Immunohistochemically stained sections of rabbit ovarian cortex with caspase 3: caspases are crucial mediators of programmed cell death (apoptosis). Caspase gives a positive reaction in the normal ovary, reacting with degenerated follicles and cells. The control group showed positive immunoreaction in growing follicles of zona pellucida, granulosa cells, and ovarian surface epithelium. In contrast, no immunoreaction in surrounding stromal cells (Figure 5A). The margarine feed group showed strong immunoreaction in growing follicles (zona pellucida, granulosa cells) and stromal cells (Figure 5B). The ghee treed group showed a moderate immunoreaction in growing follicles (granulosa cells), zona pellucida, and mild in surrounding stromal cells (Figure 5C). the olive oil feed group showing mild immunoreaction in growing follicles (zona pellucida), granulosa cells, and stromal cells (Figure 5D).

## 4. Discussion

Recently, dietary fat and oil are the key micronutrient sources that provide the human metabolic process with energy and fat-soluble vitamins [30], but they can also be dysregulated and contribute to diseases such as obesity, diabetes, cardiovascular disease, and cancer [31,32].

Our study showed that supplementation of rabbits with margarine significantly increased lipid profile (TAG, LDLc, VLDL, and cholesterol) and decreased HDL concerning control. Previous studies found that trans fatty acid consumption had been linked to serious biochemical problems, such as an undesirable serum lipid profile [33,34]. Most TFAs have physical properties similar to SFAs [35]; they increase LDL as much as SFAs but lower HDL [36]. Myristic, palmitic, and stearic acids are the three main SFAs in margarine. Palmitic acid is the most significant and can increase the production of cholesterol more than other dietary FAs [37,38]. In contrast, adding olive oil to the diet significantly decreased serum lipid profile and increased HDLC compared to the control. This is due to the high content of MUFAs, such as oleic acid in olive oil. MUFAs are suggested to be effective in improving serum lipid profile [39,40].

Furthermore, we found that the ghee-treated group had a lower lipid profile than the margarine-treated group; these results are in agreement with Nour et al. [41] and Abo-Ghanema et al. [42], who reported that ghee improves lipid profiles because of its high conjugated linoleic acid (CLA) content that decreases serum LDL in rats. Additionally, ghee is a good source of short-chain saturated fatty acids, which are easier to be digested [43] that absorbed directly into the bloodstream without being packaged into lipoproteins and are then transported to the liver as they do not need bile or pancreatic enzymes to break down. In addition, their short carbon chain fatty acids are of low calorific value and provide quick energy [44]. Conversely, Al-Othman [45] stated that ghee significantly increased serum total cholesterol levels due to its high content of saturated fatty acids.

Antioxidants produced by enzymes are crucial for protecting against cellular damage caused by oxygen metabolism. The two enzymes that catalyze the formation of hydroxyl ions and superoxide ions, respectively, are CAT and SOD. Hydroxyl radicals are produced when these antioxidants are less active, which subsequently starts and spreads lipid peroxidation [46].

Our data revealed that rabbits supplemented with margarine exhibited a significant decrease in SOD and CAT activity and significantly increased ovarian MDA levels compared to the control. These findings are in harmony with [33], which found that margarine induces a significant decrease in some antioxidant enzymes in rats indicating increased oxidative stress, and this may be attributed to the incorporation of trans fats into the cell membranes, reduce membrane fluidity, and the cells do not act as well with a subsequent product of more reactive oxygen species, which explains the increase in lipid peroxidation and a significant decrease of the total antioxidant capacity [47,48]. Moreover, Matuszewska et al. [49] indicated that increasing dietary cholesterol caused a significant decrease in GSH and SOD activities and a highly significant increase in MDA. 

On the other hand, we found a non-significant increase in catalase and SOD activities, and a significant decrease in ovarian MDA levels in the olive oil-treated group, as compared with the control. This finding is in agreement with [50], who reported that the positive antioxidant effects of olive oil against induced oxidative stress in rat ovaries might be attributed to its richness in phytochemicals that could be potential protective agents against free radicals, also the positive effect of olive oil may be due to its richness in MUFA, mainly oleic acid which has different effects on lipid profile levels and peroxidation [51]. as well, the antioxidant effect of olive oil has long been associated with the presence of caffeic acid which is famous for scavenging superoxide anions [52]. Additionally, there was a non-significant increase in CAT and SOD activities and a non-significant decrease in ovarian MDA levels in the ghee-treated group as compared with the control group. In [12], Nour, Eldin, Abd Alla, and Abd Elhady [41], ghee increased SOD and CAT activity and decreased oxidative stress and lipid peroxidation, as ghee contains CLA, which reduces oxidative stress, and lipid peroxidation improves the antioxidant system and protects the cells against oxidants [53,54]. The antioxidant effect of ghee is due to its high content of vitamins A and E, sulfur-containing amino acid (cysteine), carotenoids, antioxidant enzyme systems such as SOD, catalase, and GPx [55]. However, ghee contains a high percentage of saturated fatty acids but does not undergo lipid peroxidation [56]. 

The female reproductive cycles function primarily by the interplay between the luteinizing hormone (LH), follicle-stimulating hormone (FSH), progesterone, and estradiol (E2). The integrity of the female reproductive organs can be assayed by the serum level of these hormones [57]. FSH is a glycoprotein released in response to the gonadotropin-releasing hormone (GnRH). Also, it acts on immature follicular cells of the ovary and induces development into mature follicles and oocytes capable of steroidogenesis [58]. The principal function of the follicle-stimulating hormone is to stimulate gametogenesis, follicular development in females, and spermatogenesis in males [14,59].

The current study exhibited a significant decrease in FSH and estrogen levels and a significant increase in prolactin in female rabbits supplemented with margarine compared to other treated groups. This finding is in harmony with [14], who reported that increased production of reactive oxygen species (ROS) and free radicals at the central nervous system level and its linked glands, such as the pituitary axis of the hypothalamus, are closely correlated with altered endocrine function. It may be a major contributor to the aging of these tissues and all organ systems [60]. Aging of the hypothalamic–pituitary axis causes a cumulative decrease in function that eventually results in endocrine deficiency [61]. According to the findings of this research, the supplementation of rabbits with margarine for two months resulted in a significant increase in lipid peroxidation levels in terms of MDA in the ovarian tissue of female rabbits. Chebab et al. [62] reported decreased CAT and SOD activities and increased MDA levels (indicators of oxidative stress), leading to decreased estradiol (E2) and FSH.

Moreover, Okuyama et al. [63] found that hydrogenated vegetable oil (margarine) decreased the production of testosterone and estrogen through decreasing gene expression of the steroidogenic acute regulatory protein (STAR). LH, FSH, and estrogen receptors were also detected in ovarian tissue using immunohistochemistry, which showed a mild immunoreaction in growing follicles in zona pellucid, but no reactivity in granulosa cells. This might be due to excessive ROS generation leading to free radical attack of membrane phospholipids [64].

Reactive oxygen species (ROS) could attack cell membranes and other cellular molecules, causing lipid peroxidation, protein oxidation, DNA damage, and caspase 3 stimulation [65,66,67,68], leading to ovarian dysfunction [69,70,71]. In the present study, caspase 3 was detected immunohistochemically in ovarian tissue. The margarine group shows strong immunoreaction in growing follicles (zona pellucida), granulosa cells, and stromal cells due to the cytotoxic activity of fatty acids that influence cell survival. Long-term accumulation of lipids may lead to necrosis or apoptosis [72]. Furthermore, MDA directly reacted with DNA, causing DNA adducts and nuclear condensation, which, together with the induced mitochondrial dysfunction, promoted apoptosis via cytochrome c release and further caspase 3 activation as detected by immunostaining [73,74].

It has been shown that oxidative stress, which results from an imbalance between ROS generation and antioxidant defense, as in the margarine-treated group, can disrupt oocyte development and reduce oocyte quality [75]. The sensitivity of oocytes to ROS is extreme [76]. Despite the ghee and olive oil-treated group, an increase in lipid profile, lipid peroxidation, and decrease in serum fertility hormone levels in the margarine group was supported by our histological analysis showing vacuolation in small and growing follicles and surrounding stromal cells.

Our results indicate that there were significant (*p* < 0.05) increase in FSH and estrogen levels in the ghee and olive oil group, respectively, accompanied by a non-significant (*p* > 0.05) increase in LH and progesterone level relative to the control group. Egba, Udom and Okonkwo [14], and Moghissi [77] reported that oleuropein aglycone in olive oil is responsible increase female fertility hormone through the enhancement of the pituitary gland by the increase in noradrenaline plasma levels; in addition, olive oil contains omega-3 and omega-6 PUFAs and MUFAs, which have a strong capability of enhancing hormonal functions by stimulating the hypothalamus–pituitary ovarian axis that is responsible for the synthesis and storage of gonadotrophins (LH and FSH), which play a major role as regulators of folliculogenesis and subsequently the fertility of females.

Moreover, ghee is an excellent source of conjugated linoleic acid (CLA) [13] that improves female fertility hormone involving improved ovarian follicular steroidogenesis and increased circulating centration of IGF-I [78]. IGF-I is important in follicular and luteal development [79], enhancing the growth, synthesis of progesterone, and LH binding sites in bovine thecal cells [80] and increasing estradiol production by bovine granulosa cells in vitro [81], as well as intrafollicular infusion of IGF-I increased the size of the dominant follicle [82]. So we found a high immunoreaction of estrogen, LH, and FSH receptors in ovarian tissue by using the immunohistochemistry in the ghee and olive oil-treated group.

In our study, the significantly increased antioxidant enzyme and decreased lipid peroxidation in ovarian tissue in the ghee and olive oil-treated groups was supported by using the immunohistochemistry of ovarian caspase 3, which showed mild immunoreaction in both groups due to positive antioxidant effects of olive oil against oxidative stress [50]. Furthermore, ghee has a high antioxidant activity, as it contains lipophilic vitamins. Ref. [83] especially vitamins A and E [84]. Vitamin E is present in all cell membranes and works hard in this lipid environment to minimize free radicals and prevent significant lipid peroxidation caused by a free radical chain reaction [85]. Moreover, it suppresses the apoptosis in the germinal epithelium [86].

The increased female fertility hormones levels and antioxidant enzyme activity in ghee and olive oil-treated groups were supported by our histological findings, which showed proliferation in follicular cells of primary follicles, normal small follicular follicles, and granulose cells of growing follicles, and this might be due to increasing FSH which mainly responsible for the oocyte growth by stimulating estradiol production and promoting, while LH mainly takes charge of the oocyte maturation [87].

## 5. Conclusions

Our results demonstrated that ghee and olive oil consumption enhanced the lipid profile, an antioxidant enzyme, decreased lipid peroxidation, and improved female fertility-related parameters. As well, as our data revealed that ghee and olive oil-treated groups showed mild immunoreaction of ovarian caspase 3, which supports the positive antioxidant effects of olive oil and ghee against oxidative stress; on the other hand, margarine is an unhealthy dietary fat source as it increases the lipid profile and lipid peroxidation, decreases antioxidant enzymes, and female fertility-related parameters. Future research must examine the molecular pathway mechanism of olive oil and ghee.

## Figures and Tables

**Table 1:** Ingredients of basal diet.

Ingredient	g	Ingredient	g
Yellow corn	7.5	Di-calcium phosphate	0.5
Wheat bran	24	DI-methionine	0.2
Barley	20	Anti-aflatoxin + anti-coccidian	0.5
Clover hay	22	Vitamin and minerals premix	0.30
Soybean meal (44% CP)	23.5	NaCl	0.35
Limestone	1.15	total	100

**Table 2:** Means and their standard errors of rabbits’ body weights (kg), ovarian MDA levels (nmol/mg protein), catalase activities (U/mg protein), SOD activities (U/mg protein) in normal (NC), margarine, ghee, and olive oil- treated rabbits at the end of the experiment.

Group	Body Weight (kg)	Oxidant and Antioxidant Markers		
		MDA (nmol/mg Protein)	Catalase (U/mg Protein)	SOD (U/mg Protein)
Control (NC)	3.9 ± 1.4 ^b^	101.3 ± 0.87 ^b^	31.7 ± 2.1 ^b^	14.7 ± 2 ^b^
Margarine	4.57 ±1.2 ^a^	145.8 ± 3.2 ^a^	18.3 ± 2.1 ^c^	8.9 ± 1.6 ^c^
Ghee	3.53 ± 1.9 ^b^	109 ± 1.8 ^b^	45.1 ± 1.8 ^a^	18.2 ± 1.2 ^a^
Olive Oil	3.75 ± 1.7 ^b^	92.9 ± 3.4 ^c^	46.5 ± 3.2 ^a^	21.3 ± 1.55 ^a^

All values are expressed as the mean ± SE. Mean values bearing different superscripts in the same column significantly differed at *p* < 0.05.

**Table 3:** Means and their standard errors of FSH (ng/mL), estradiol (E2) (pg/mL), LH (mIU/mL), progesterone (ng/mL), prolactin (pg/mL), and testosterone (ng/mL) levels in normal (NC), margarine, ghee, and olive oil-treated rabbits at the end of the experiment.

Group	Fertility Hormones					
	FSH	Estradiol (E2)	LH	PROGES	PRL.	Testosterone
Control	6.6 ± 0.4 ^b^	77.1 ± 3.7 ^b^	4.8 ± 0.4 ^a^	1.84 ± 0.15 ^a^	16 ± 1.6 ^b^	1.38 ± 0.03 ^a^
Margarine	5.1 ± 0.2 ^c^	68.5 ± 1.5 ^c^	4.6 ± 0.12 ^a^	1.78 ± 0.09 ^a^	31 ± 2.5 ^a^	1.32 ± 0.08 ^a^
Ghee	9.3 ± 0.6 ^a^	88.4 ± 1.6 ^a^	5.09 ± 0.88 ^a^	2.1 ± 0.22 ^a^	14.5 ± 0.7 ^b^	1.41 ± 0.04 ^a^
Olive Oil	8.7 ± 1.4 ^a^	82 ± 4.5 ^a^	4.9 ± 0.33 ^a^	1.9 ± 0.05 ^a^	13.7 ± 1.2 ^b^	1.49 ± 0.01 ^a^

All values are expressed as the mean ± SE. Mean values bearing different superscripts within the same column significantly differed at *p* < 0.05.

**Table 4:** Means and their standard errors of serum TAG (mg/dL), cholesterol (mg/dL), HDL (mg/dL), LDL (mg/dL), VLDL (mg/dL), ALT (U/L) and creatinine (mg/dL) levels in normal (NC), margarine, ghee, and olive oil-treated rabbits at the end of the experiment.

Group	TAG	CHOL.	HDL	LDL	VLDL	ALT	Creatinine.
Control (NC)	201.3 ± 2.9 ^a^	143.3 ± 9.3 ^b^	37 ± 2.1 ^b^	66 ± 3.5 ^b^	40.26 ± 0.6 ^a^	43.7 ± 4.2 ^b^	0.89 ± 0.05 ^b^
Margarine	204.8 ± 6.1 ^a^	173.2 ± 3.8 ^a^	26.7 ± 1.7 ^c^	105.5 ± 4.7 ^a^	41 ± 1.2 ^a^	55.8 ± 1.3 ^a^	1.3 ± 0.01 ^a^
Ghee	171.5 ± 5.8 ^b^	146.4 ± 3.1 ^b^	35.5 ± 2.2 ^b^	76.6 ± 4.1 ^b^	34.3 ± 1.2 ^b^	46.6 ± 1.8 ^b^	1.02 ± 0.04 ^b^
Olive Oil	148.6 ± 5.7 ^b^	119.3 ± 2.7 ^c^	48.5 ± 1.5 ^a^	41.1 ± 3.1 ^c^	29.7 ± 1.1 ^b^	44 ± 2.2 ^b^	0.92 ± 0.02 ^b^

All values are expressed as the mean ± SE. Mean values bearing different superscripts within the same column significantly differed at *p* < 0.05.

## Data Availability

The data can be shared up on request.

## Supplementary Materials

The following supporting information can be downloaded at: https://www.mdpi.com/article/10.3390/life13010080/s1, Table S1: Fatty acids composition of margarine, ghee and olive oil.
