# Supplementary material for: Natural Ghee Enhances the Biochemical and Immunohistochemical Reproductive Performance of Female Rabbits
Source: Life (Basel). 2022 Dec 27;13(1):80. doi: 10.3390/life13010080 (PMC9861198; doi:10.3390/life13010080)
Supplement: Supplementary file 1 [file life-13-00080-s001.zip › life-2013070-supplementary.pdf]

Table S1: Fatty acids composition of margarine, ghee and olive oil.

Fatty acids	Margarine	Ghee	Olive oil
Butyric acid C4	0	2.25	0
Caproic acid C6	0	1.22	0
Caprylic acid C8	0.01	0.59	0
Capric acid C10	0.02	1.24	0
Lauric acid C12	0.25	1.95	0
Myristic acid C14	1.15	11.14	0.21
Myristoleic acid C14	0	0.22	0
12- methylemyristic acid C15	0	8.5	0
Pentadecanoic acid C15	0.03	2.09	0
Palmitic acid C16	36.98	31.45	15.5
Margaric acid C17	0.09	1.34	0.01
14-methylpalmitic acid C17	0	0.53	0
Stearic acid C18	5.01	18.47	5.1
Glycidyl palmitate acid C19	0.44	1.74	0.21
Glycidyl oleate acid C21	0.36	2.45	0.11
**Saturated fatty acids**	**43.34**	**77.53**	**21.14**
Palmitoleic acid C16:1	0.1	1.33	2.3
Oleic acid C18:1	44.72	18.53	68.5
**Monounsaturated fatty acids**	**44.82**	**32.8**	**70.8**
Linoleic acid C18:2	1.48	1.53	8.2
Linolenic acid C 18:3	0.15	0.87	0.4
Conjugated linoleic acid C18:2	0.0	0.21	0.01
**Polyunsaturated fatty acids**	**10.86**	**2.61**	**8.61**

Fatty Acid Composition

Fatty acid composition analysis of margarine, ghee and olive oil were done at the Agriculture Research Center, Doky, Giza.

1.Preparation of fatty acid methyl esters:

The fatty acid methyl esterswere prepared using transesterification with cold methanolic solution containing potassium hydroxide as catalyst. The fatty acid methyl esters were identified by G.C capillary column according to the methods of IOOC (2001).

2.Identification of fatty acid methyl ester by GLC:

Agilent 6890 series GC apparatus provided with a DB-23 column (60mx 0.32 x 0.25µm). Fatty acid results after the previous procedure steps were transformed into methyl esters and directly injected into the GC. Carrier gas was N2with flow rate of 2.2 ml/min, splitting ratio of 1:80.The injector temperature was 270°C. The temperature setting were as follow :130° to 210°C at 3min, and held time 10 min. at 210°C.
